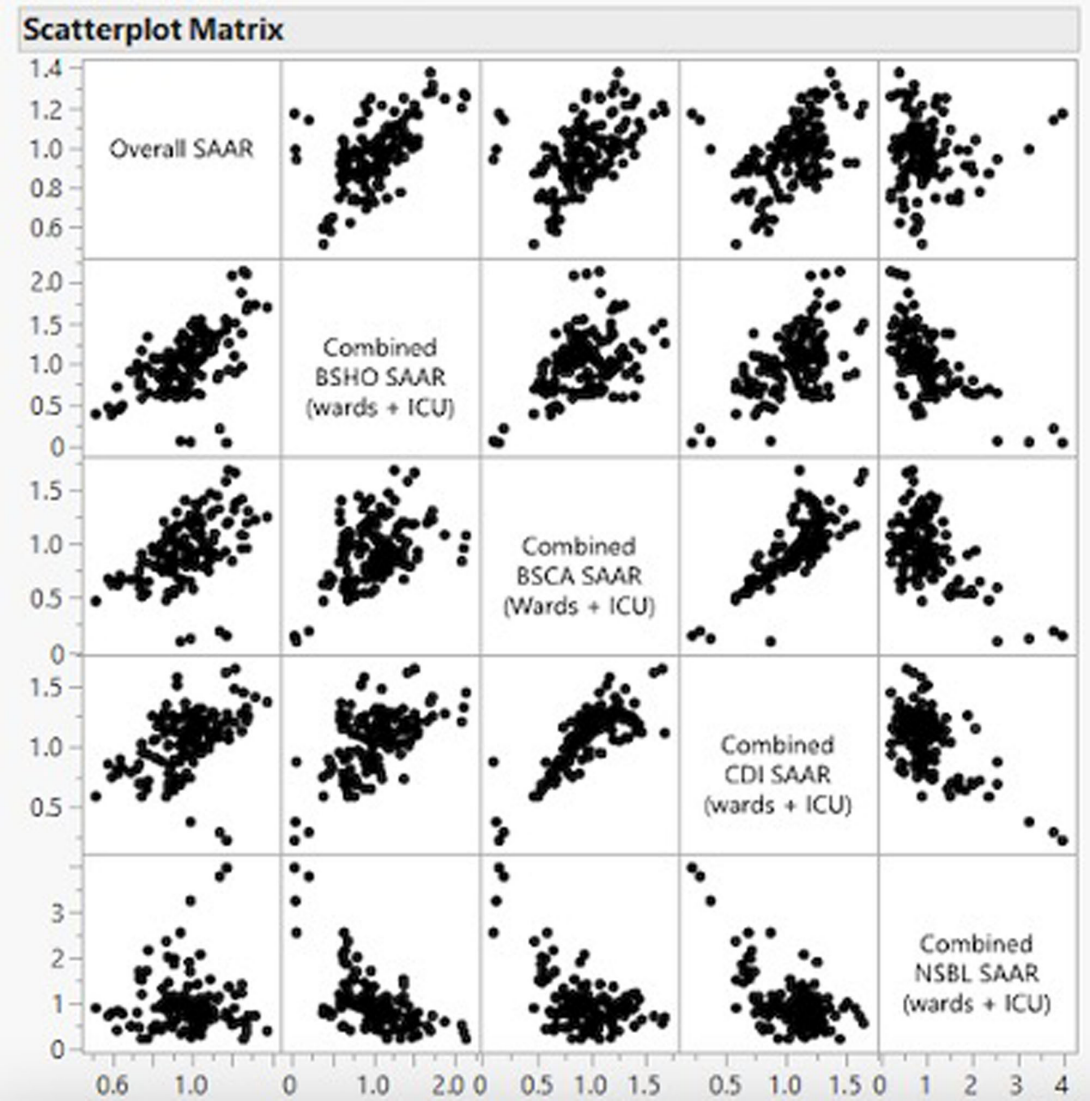# Understanding the Impact of Narrow Spectrum Beta-Lactam Use on Overall and Broad-Spectrum Antimicrobial Utilization in South Carolina

**DOI:** 10.1017/ash.2024.172

**Published:** 2024-09-16

**Authors:** Kayla Antosz, Sarah Battle, Pamela Bailey, Hana Winders, Majdi Al-Hasan

**Affiliations:** ASC-SC, University of South Carolina College of Pharmacy; University of South Carolina; Prisma Health-Midlands; Brandon Bookstaver; University of South Carolina School of Medicine

## Abstract

**Background:** The standardized antimicrobial administration ratio (SAAR) is a metric utilized to measure antimicrobial use within and between hospitals by comparing observed to predicted antimicrobial days of therapy. However, it remains unknown whether narrow-spectrum beta-lactam (NSBL) use adds to overall antimicrobial utilization or substitutes broad-spectrum agents. This muti-hospital cohort study examined the impact of NSBL use on overall antimicrobial utilization and the correlation between the use of NSBL and various broad-spectrum antimicrobial categories in South Carolina (SC) hospitals. **Methods:** SAARs were collected from all hospitals in SC that reported antimicrobial use (AU) data to the National Healthcare Safety Network (NHSN) between 2017 and 2021. SAARs collected included: overall SAAR, broad-spectrum agents predominantly used for hospital-onset infections (BSHO), broad-spectrum agents predominantly used for community-acquired infections (BSCA), NSBL, and antibacterial agents posing the highest risk for Clostridioides difficile infection (CDI). Category SAARs were combined to include data in both the adult intensive care unit (ICU) and adult wards using the formula: [(total observed antimicrobial days ICU + total observed antimicrobial days ward) / (total predicted antimicrobial days ICU + total predicted antimicrobial days wards)]. Pearson correlation coefficient (r) was used to examine the correlation between various SAARs categories. **Results:** A total of 38 hospitals in South Carolina reported AU to NHSN at least during one calendar year during the study period. The use of NSBL agents was negatively correlated with the use of BSHO (r = -0.596, p < 0.001), BSCA (r = -0.543, p < 0.001), and high-risk CDI antibiotics (r = -0.601, p < 0.001). Moreover, the use of NSBL agents did not correlate with the overall SAAR (r = 0.008, p = 0.93), whereas the use of BSHO (r = 0.587, p < 0.001), BSCA (r = 0.494, p < 0.001), and high-risk CDI agents (r = 0.464; p < 0.001) positively correlated with the overall SAAR. **Conclusion:** In South Carolina hospitals, the use of NSBLs does not contribute to additional antibiotic use overall as it seems to replace broad-spectrum antimicrobials in various categories. By de-escalating from broad-spectrum agents to NSBL agents, one can improve antimicrobial use without negatively impacting the overall SAAR. This observation encourages implementation of antimicrobial stewardship interventions that increase utilization of NSBLs, when appropriate, such as de-escalation of antimicrobial therapy among others without concerns for increasing the SAAR for overall antibiotic use.

**Figure:**